# Implementation and Early Impacts of an Integrated Care Pilot Program in China: Case Study of County-level Integrated Health Organizations in Zhejiang Province

**DOI:** 10.5334/ijic.5605

**Published:** 2021-08-30

**Authors:** Meng Jia, Fang Wang, Jiangen Ma, Miaomiao Tian, Minjie Zhao, Liming Shen

**Affiliations:** 1 Institute of Medical Information, Chinese Academy of Medical Sciences, Beijing, CN; 2 Health Commission in Huzhou, Zhejiang, CN; 3 Medical Administration Department of Health Commission in Deqing County, Zhejiang, CN

**Keywords:** integrated delivery network, regional healthcare, accountable care organization, referral system

## Abstract

**Background:**

One of the most noticeable integrated care-related policies in China is the growth and proliferation of County-level Integrated Health Organizations (CIHOs), which take over a set of primary healthcare institutions to form an integrated delivery network in order to achieve an ordered hierarchical delivery system by strengthening primary care.

**Objectives:**

This paper presents emerging findings from an ongoing evaluation of the early impacts of the demonstrator site, Deqing CIHO, in Zhejiang Province, in order to examine the extent to which the implementation has achieved its core objectives: (1) establishing the hierarchical referral system, (2) capacity building of primary healthcare providers, and (3) reducing the costs.

**Design:**

This case study was conducted to determine institutional and managerial processes.

**Settings:**

Data were collected and analyzed at the CIHO and county level. A structured questionnaire was used for data collection.

**Primary and secondary outcome measures:**

Indicators were selected from the existing database of the county health system and arranged into three segments to assess (1) service utilization among each level of care; (2) capacity-building progress for primary care centers, (3) cost-related indicators for both levels of care.

**Results:**

Service utilization data show that one year after CIHO implementation, the proportion of patients who chose to get inpatient care outside of the county decreased from 27.3% to 24.5%. Hospital admissions were retrieved from outside the county, while service volume slightly shifted from hospitals to primary care sites. Capacity-building indicators for township health centers show that 6 out of 12 items showed better performance compared to the national average growth rate, and a moderated growth rate appeared in terms of per capita cost.

**Conclusion:**

Progress evaluation results from Deqing CIHO indicated some positive effects on three main outcomes, which reveal the potential of CIHOs in not only strengthening primary care but also controlling cost as a result of early implementation. Further emphases of evaluation are required to determine the impacts on the quality and experience of care that are estimated using claim-based data at the individual level.

## Introduction

Primary healthcare providers in China are not necessarily the point of first contact; therefore the entire delivery system lacks the crucial role of gatekeeper. Of all the outpatient visits across the country in 2018, only 53% occurred at primary healthcare sites compared to 62% in 2010; this decline has been consistent [1].

The current system of health-care delivery in China is fragmented, hospital-centric and treatment-dominated, with little effective collaboration among institutions in different tiers of the system [2]. To counter the rising burden of a hospital-centric delivery system, China has introduced a series of polices aiming to facilitate a rational referral system, with integrated care-related policy being the focus of the moment. Over the last decade, integrated care has been suggested as one strategy for promoting coordinated health-care delivery, improving quality of care and reducing costs [3]. In 2016, the report of *Deepening Health Reform in China* was published jointly by the WHO, the World Bank and the Chinese government [2]. The report proposed strengthening health care in China through a tiered health-care delivery system in accordance with a people-centred integrated care model. Despite the international trend of integrated care reforms, which commonly focus on improving care coordination and quality as well as lowering the expenditure [4], China’s integrated care strategy was dedicated to address the main issues in the delivery system, which are the increasingly concentrated attendances in hospitals, as well as the inadequate capacity and use of primary healthcare providers [5].

### Development of integrated care models in China

Political support for integrated care is strong and has continued over time. Integrated delivery of healthcare is currently at the forefront of healthcare policy. The movement toward an Integrated Delivery System (IDS) in China is referred to as Integrated Health Networks (IHNs), in which the government plays an active role in organizing different levels of providers (tertiary, secondary, and primary). This movement has overlapped with another national-scale integration-related strategy—the Family Physician Registry Campaign—since 2016. These two innovations birthed via separate movements were recognized as the “toolbox” for the redesign of primary care in order to achieve the ultimate goal of an ordered referral system in China.

The IHN Pilots Program [6] was launched on a scattered basis in 2017, followed by a National Integrated Care Model Strategic Plan [7] released by the State Council General Office. According to the guidelines of the strategic plan, system-wide models of integrated care are created as designated ways to facilitate diverting patients from tertiary hospitals to primary care settings. Differential strategies for the integration were applied due to the significant urban–rural disparity (see ***Table 1***).

**Table 1:** Differential strategies for integration in urban and rural areas.


	MEDICAL GROUPS	CIHOS	ADVANCED CIHOS

**Coverage**	Urban	County regions (rural)	County regions (rural)

**Model composition**	1) Hospital and community health centers within a specific geographical area; 2) community health centers that are affiliated	County hospital, township health centers, and village clinics within catchment area	Same as CIHOs

**Model structure**	1) Vertical technical support; 2) horizontal integration of organizations	Vertical integration of organizations	Vertical integration of organizations

**Accountable for population health**	1) No; 2) yes	No	Yes

**Payment models**	Fee-for-service	Fee-for-service	Shared savings


In urban areas, each tertiary hospital establishes relationships with primary health centers within a specific geographic area on a contract basis, forming a Medical Group. Although the Medical Group is ruled by an integrated strategic plan, services provided by hospital and primary care institutions are still on a separate basis and there are no shared savings or risks between these two levels of providers. By the end of 2019 all the tertiary hospitals across the counties had participated in the initiatives.

Most of the counties in China are rural and underdeveloped areas, accompanied by the shortage of various resources, including medical resources. The development of county-level integrated health organizations (CIHOs) is one of the priorities of medical reform in China, which has provided great supports in the medical aspects for areas with limited medical resources. In rural areas, CIHOs were promoted by merging county hospitals and primary healthcare institutions under a single organization. County hospitals were matched with the township health centers (THCs) within their geographical area of influence. The number of CIHOs varies in different counties, ranging from 1 to 4. The aim of the CIHOs strategy is to lower hospital-related utilization while promoting the capacity and utilization of primary healthcare providers. It is designed to cover all the 700 million people living in county areas, or approximately half of China’s population.

The national strategy on CIHOs has evolved over time. As an improved version, the advanced CIHO pilots program [8] was launched on a national scale in early 2019, followed by a detailed implementing guideline [9] serving as the instrument for the model development at the macro and micro levels. CIHOs began to emerge in a large scale, with 567 pilot programs launched across the country by the end of 2019.

Advanced CIHOs have been proposed as an important strategy for achieving the triple aim of a health delivery system at the county level that optimizes regional healthcare in the following ways: (1) capacity building of primary healthcare providers; (2) reducing health expenditure; and (3) ultimately, establishing an ordered referral system in which health service is organized in regional hierarchical networks. To practice of accountable care reform in China, advanced CIHOs are population-based models characterized by their accountability for public health and shared savings.

**Figure 1:**
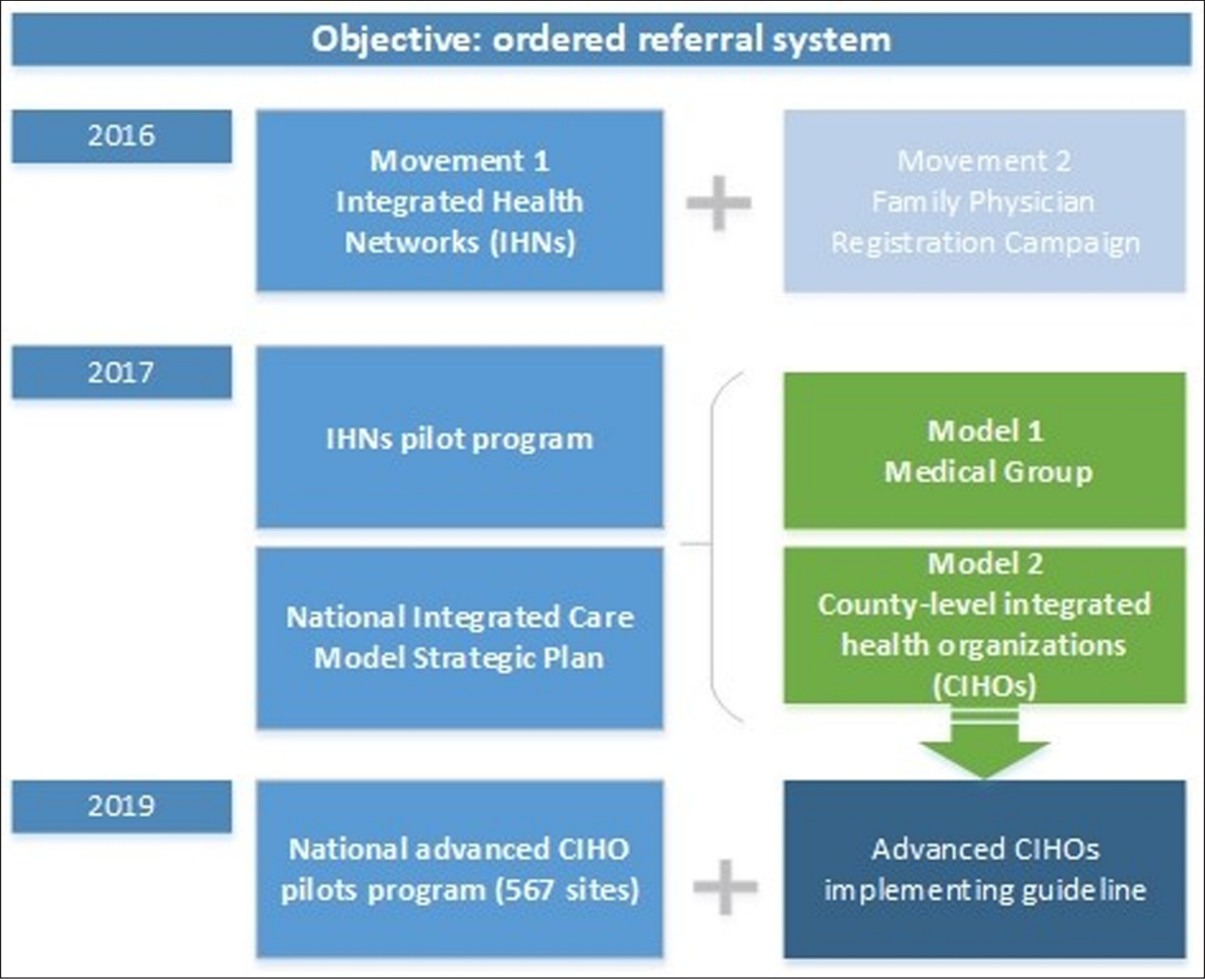
Roadmap of the development strategies for integrated care models in China.

The roadmap for the development of integrated care strategies is demonstrated in the flow chart in ***Figure 1***. The advanced CIHO model is the focus of this study. The purpose of this case study is to describe the CIHO model components and innovation activities, estimate its early impacts, and summarize the key lessons that have been learned from the early performance years of the pioneer CIHO model in China.

## Pilot project of county-level integrated care in Zhejiang province

Policy-related context regarding the development and progress of integrated care in Zhejiang has been recognized as enabling and mature [10], influencing the rest of the country. To follow the national guidance of promoting an integrated care strategy, the CIHO Demonstrator Project in Zhejiang Province was launched in 2017, and demonstrator sites were identified within 13 counties in the province [11]. After one-year of practical exploration, a fully implementable plan [12] was released by the provincial government, aiming at expanding the existing experiences beyond the pilot phase. The key commitments of this plan were the transformation and upgrading of the care delivery model and institutional innovations in the administrative and operational mechanisms of the health system at the county level. To fulfill the objectives of the plan, an official to-do list was issued, which identified 55 priority tasks for developing new organizational solutions.

By the end of 2019, a total of 161 CIHOs were created across all of the 70 counties in the province. Plans are in place for the entire province to be covered by CIHOs by the end of 2020.

## CIHOs in Deqing County

**Figure 2:**
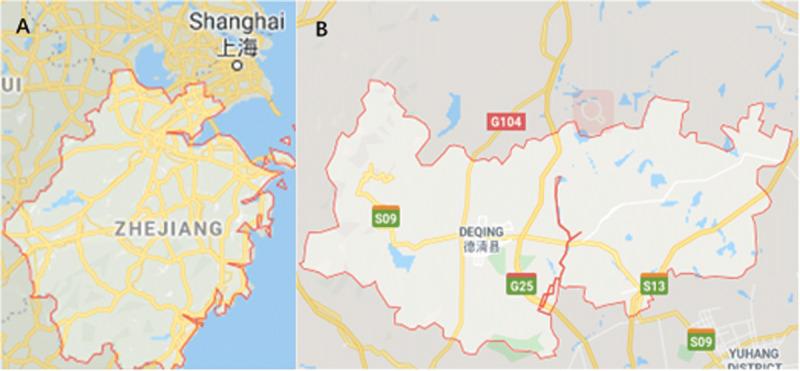
Map of Deqing county (**B**, bright area), within Zhejiang province (**A**, bright area).

**Figure 3:**
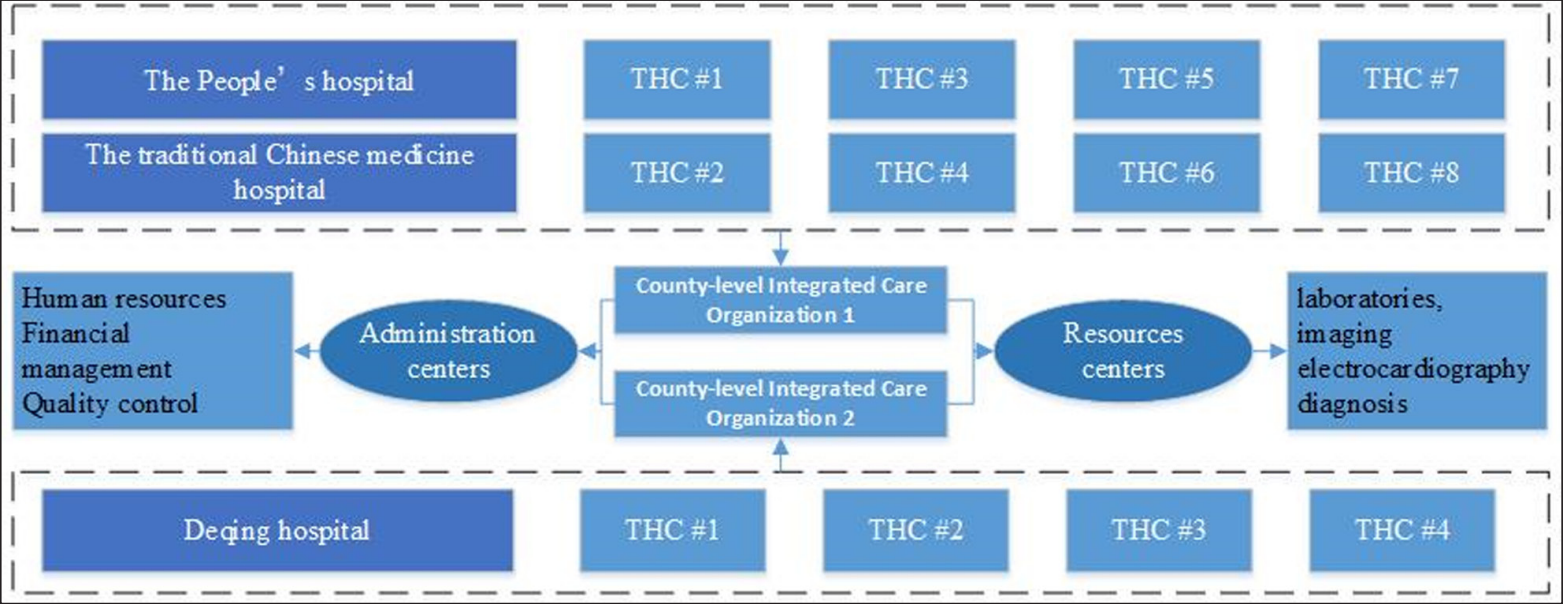
The organization chart of CIHOs in Deqing to demonstrate the relationships among the medical institutions and resources centers.

Deqing County, the map of which is shown in ***Figure 2***, is one of first 13 demonstrator sites in Zhenjiang Province, initiated in September 2017. It was considered one of the most prominent CIHO pilots in China. Two CIHOs (***Figure 3***) were created in the area, with a catchment population of around 442,000 residents. One comprised two county hospitals (the People’s hospital and the traditional Chinese medicine hospital) and eight THCs; the other operated on a smaller scale, comprising the Deqing Hospital and four THCs. The main dimensions of intervention and approaches to integration that have been employed by the pilot program include structural, functional, clinical, and service integration, as well as the payment models.

### Organizational integration and the governance structure

Committees were established for each CIHO, usually comprising members from both THCs and the county hospital. They met regularly and were operating under the supervision of the County Health Department. While the legal status of each THC remains independent, the legal representative is replaced by one from the county hospital, most commonly the executive director of the hospital. By taking over a set of THCs, the county hospital centralizes the power of governance.

### Functional, Clinical, and Service Integration

With regard to functional integration, regional hubs for laboratories, imaging, and electrocardiography diagnosis were restructured in an integrated manner; consequently, the inter-accreditation between THCs and the county hospital for testing results is guaranteed so as to avoid duplicate testing for the patient.

Communication about information systems between two levels of care is a major obstacle during the integration process due to the absence of a unified information platform in primary care settings. As a pioneer, Deqing CIHO developed its own information system for use among primary health centers, the HIS (Hospital Information System) for Primary Care, making it possible for electronic medical records to be shared between the county hospital and the THCs that depend on it.

To strengthen the coordination of care across a continuum of services and settings, the Continuous Healthcare Center was instituted at the county hospital, which undertakes the roles of managing referrals, running preadmission tests, and ensuring follow-ups after discharge.

### Payment model

Prospective payment reform has been proposed as the most prominent character of CIHOs. Deqing CIHO adopted several payment options including shared savings and blended payment for primary care. Each CIHO is accountable for the total cost of care for their assigned beneficiaries (regional-based), even if the care is provided outside of the organization. The system also includes an enhanced payment for Family Physician Registry in the form of a per-member-year care management fee in addition to traditional fee-for-services payments.

## Evaluation of the early impacts in deqing county

An early-stage evaluation program was commissioned by our research team as a preliminary assessment of a large-scale national evaluation of CIHO pilot programs initiated by the Primary Health Department of the National Health Commission. The evaluation focused on the institutional and managerial process, rather than specific clinical influence on health outcomes due to the short time of implementation (less than three years by the time of this evaluation). Therefore, data on public health status or experience of care will not be included in this study. Evaluation activity was conducted to exam the extent to which the implementation has achieved its core objectives, which are: (1) establishing the hierarchical referral system, (2) capacity building of primary healthcare providers, and (3) reducing costs.

### Evaluation tool

A structured questionnaire was used to collect data. For the purpose of the study, we identified measures to reflect the three aims established above, which were also linked to the existing local plan for performance evaluation. Indicators were selected from the existing database of the county health system and arranged into three segments to assess (1) service utilization among each level of care; (2) capacity-building progress for primary care centers, (3) cost-related indicators for both levels of care. Criteria for indicator selecting are a) being consistent with the established aims of the pilot, b) being part of the existing local plan for program performance evaluation, and c) being available to be extracted from the existing database of the county health system, instead of creating a new one.

### Data sources

Utilization and insurance data were all collected at the county level. Yearly aggregate information was used collectively so that no individual could be identified by such compiled information. Service-utilization related data were extracted from the administrative information system of County Annual Health Statistics with authorization by the County Bureau of Health. Insurance-related data were provided by the County Medical Insurance Office and were aggregated in a way to ensure that they answered the evaluation questions. Capacity-related data were collected at the organization level. Because Deqing CIHO was established in 2017, research data were collected from 2016 to 2018, with a one-year interval before and after the starting point.

## Results

### Service utilization

In order to achieve the objective of the hierarchical referral system, two aspects were expected to show shifting patterns of service utilization, which are presented as the hypotheses of this study:

1. Service utilization was maintained within the county to achieve higher patient containment (patients are less likely to seek medical service outside of the network).
2. Attendances shifted from county hospital to primary health centers.

**Figure 4:**
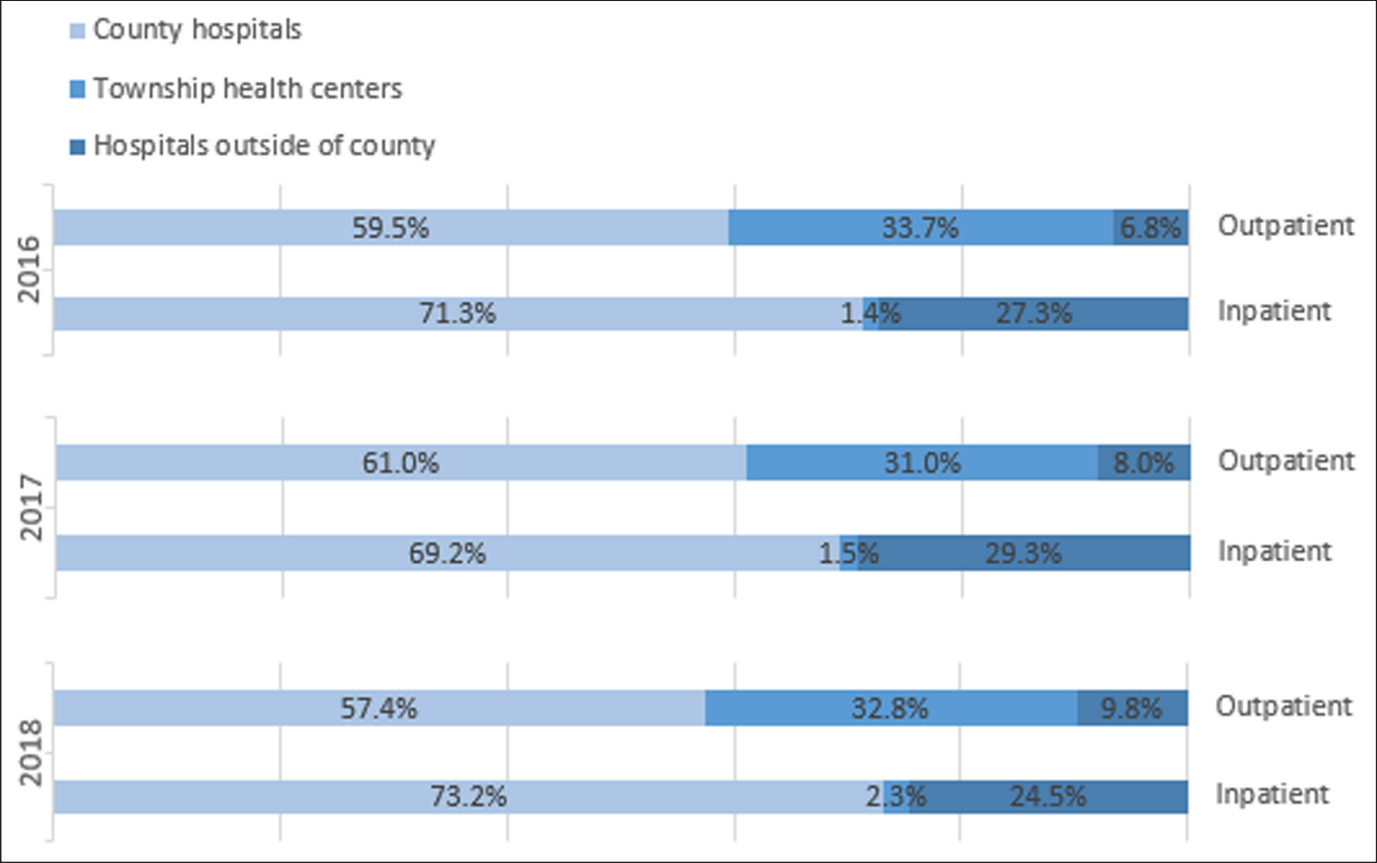
Service utilizations among three levels of care providers.

The volume of service utilization for inpatients and outpatients are monitored at three levels of care providers. As shown in ***Figure 4***, there is a growing trend of patients seeking outpatient care outside of the county. The percentage of outpatient services in hospitals outside of the county has been growing continually from 6.8% in 2016 to 9.8% in 2018. However, the upward trend of hospitalizations outside of the county has been reversed to some extent. The drop in the proportion of inpatient care occurred outside of the county (from 27.3% in 2016 to 24.5% in 2018) and was considered to be remarkable, considering the fact that as many as 30% of patients chose to be hospitalized outside of the county in 2017. At the CIHO level, higher patient containment was achieved in term of inpatient care. The gains in inpatient containment were maintained in both county hospitals and THCs; however, the latter has shown potential of gaining more power, given that the percentage of THC hospitalizations increased from 1.4% in 2016 to 2.3% in 2018.

**Figure 5:**
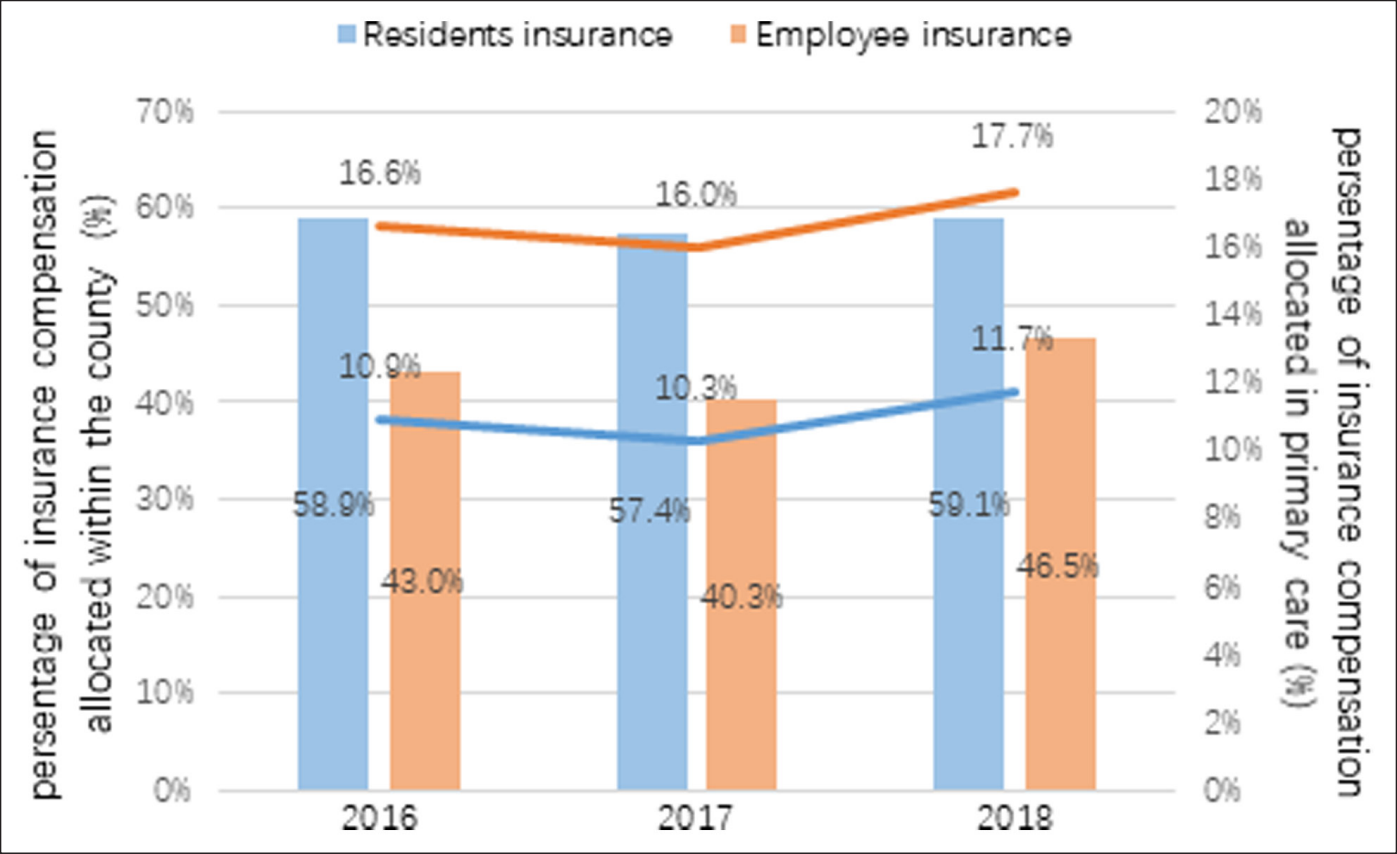
Medical insurance compensation allocation in Deqing County (bars represent the compensations within the county, lines represent the proportion of primary care).

Although not the solid evidence, of transition in service utilization, allocation of medical insurance compensation is demonstrated in ***Figure 5*** as a secondary proof. The proportions of compensation that are allocated within the county and at primary care sites appear to indicate a modestly growing tendency, which suggests that hospital admissions were retrieved from outside the county while service volumes were simultaneously shifting from hospitals to primary care sites.

### Capacity-building indicators

To access the effects on capacity-building progress of THCs, 12 indicators in two dimensions were selected from the existing annual health statistic report system, including eight items related to the core capacity indicators, and four items related to financial performance. As shown in ***Table 2***, the number of general physicians (GPs) increased (above the National average growth rate), and both the per capita number and the GPs/specialists ratio showed a marked increase. Evidence supports the view that THCs in Deqing were running on a more active and productive basis, as the two medical efficiency indicators (occupancy rate for curative care beds and the average length of stay) were superior to those of the national average. Further, the financial indicators including total revenue, personnel cost, and medical income per physician showed a notably increasing trend.

**Table 2:** Changes in capacity-building indicators of township health centers in Deqing County, 2016–2018.


	2016	2017	2018	AVERAGE ANNUAL GROWTH RATE	NATIONAL AVERAGE GROWTH RATE*

**Capacity indicators**					

Number of GPs per 10,000 people	1.04	1.41	1.63	25.2%	21.3%

GPs/specialists ratio	0.36	0.53	0.55	23.1%	–

Nurses/doctors ratio	0.45	0.47	0.43	–3.0%	–

Number of beds (per THC)	86	158	155	34.3%	–

Average length of stay	10.4	7.5	6.6	–20.3%	0

Occupancy rate for curative care beds	16.5%	20.8%	22.8%	17.5%	–0.8%

Disease spectrum in ambulatory care	62.2	70.67	74.3	9.3%	–

Disease spectrum in hospitalizations	5.4	7.1	10.78	41.3%	–

**Financial indicators**					

Total revenue (per THC, in 1,000,000 CNY)	17.40	19.85	24.25	18.1%	9.9%

Proportion of consultation fee to total income	17.8%	18.0%	21.5%	9.9%	

Proportion of personnel cost to total cost	35.6%	38.9%	37.4%	2.5%	1.5%

Professional income per physician (in 10,000 CNY)	51.65	124.69	120.41	52.7%	6.1%


* Source: *National Healthcare Annual Statistics*, 2017–2019.

Furthermore, indicators for disease spectrum in both ambulatory care and hospitalizations were captured via the HIS for Primary Care system and were computed to an average level of THCs, suggesting the improvement in care capacity of primary care, especially for those with diseases requiring hospitalization (***Table 2***).

### Per capita costs

**Figure 6:**
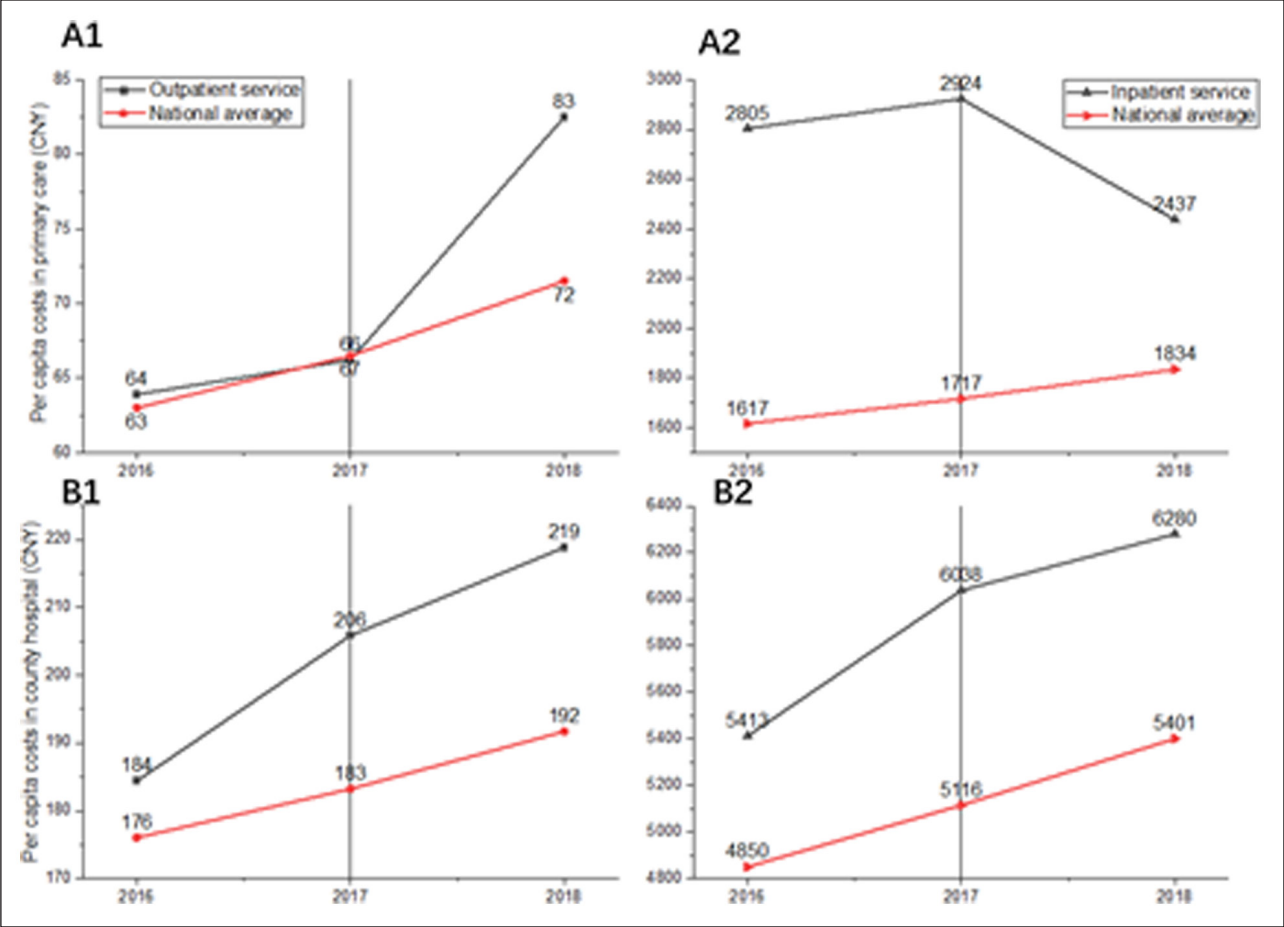
Per capita costs in two levels of care (**A** for township health centers, and **B** for county hospitals) in Deqing CIHO.

Due to the fact that THCs and county hospitals have been running separately in term of payment mechanism, cost-related data were acquired independently from these two levels of care providers. The per capita costs in Deqing CIHO are generally above the average spending for health in China’s county regions, as demonstrated by the red lines in ***Figure 6***. As we can tell by the chart of trends, changes in costs across the two levels of care are inconsistent. In primary care settings, a decreasing trend in inpatient costs provided by THCs was detected (A2), whereas the per capita for outpatients was increasing substantially (A1). In the county hospital, per capita costs for both outpatient and inpatient care are increasing, although a moderated growth rate has appeared since 2017 (B1 and B2). The finding of a decreased (A2) as well as decelerating trend (B1 and B2) in per capita costs may suggest that although the CIHO model has slowed the growth of health spending at the county level, there is no evidence of cost-saving effects that can be directly attributed to CIHOs.

## Discussion

The theory and practice of organizational design for an IDS have been thoroughly demonstrated around the world over the last decade [13,14,15,16,17]. There is plenty of evidence and guidance for policymakers on how to design and implement an IDS. Counties and local implementing experiences may serve as useful models; for instance, the Integrated Health Organizations (IHOs) in Basque County in Spain [18] and the Regional-Based IHNs in Brazil [19]. A comparison of international IDS models reveals that they share similar structures and characteristics, being government-facilitated, vertical, and population-based integrated care models, offering potentials for learning in a China setting.

In the case of Deqing, when adopting a CIHO model in practice, a government-facilitated top-down approach was applied. In the first two years of the pilot program, implementation primarily focused on the organization restructuring process, rather than service and clinical integration. Although specialists from county hospitals were assigned to each THC to provide technical support, the actual workflow interaction was rare between primary health providers and specialists. It was observed that under a broad-ranging integration framework, nested models of care—for instance, the coordinated clinical pathways designed for specific-disease groups—were not in place yet. In the context of domestic literature, numerous research projects were conducted to study the configuration of organization itself, but few focused on clinical integration at the provider level. So far there is no existing evidence of Deqing CIHO providing better coordinated care to patients. This raises the concern that many pilot programs were integrated only by merging their organizations but not to provide better coordinated care to clients [20]. As demonstrated in other studies, organizational and functional integration neither guarantees clinical integration at the provider level, nor is sufficient to achieve it [21]. For a CIHO being able to offer true coordination and continuity of care, further emphasis is required on accountable care and service and clinical integration.

With regard to the role of primary care, our findings corresponded to those of a study on IHOs in Basque County in Spain [22]. Both found that the budget allocation was slowly moving from specialized care to primary care. This transition indicates that primary care is gaining more power in the scenario of regional-based integrated care models. The observed improvements in capacity-building indicators in THCs suggest that CIHOs may have the potential to reverse the trend of declining utilization and capacity of primary care in Chinese counties.

One of the most pressing concerns is that instead of reactivating and strengthening primary care, the existing regional resources and powers were shifting to and were further centralized in the county hospital, which treated the integration as an opportunity to expand itself. The creation of CIHOs actually accelerated this process, acting in an opposing way to the stated objective of the CIHO strategy. This study revealed that hospitalizations showed a slight trend from county hospitals to the THCs, but no obvious changes were observed in terms of outpatient care. There is still a chance that county hospitals were taking over THCs in order to expand the volume of inpatient services. To avoid this adverse effect, on the one hand, policymakers aim to entice payers to move away from fee-for-service systems, toward bundled payment systems that reward coordinated and high-value care. On the other hand, as required by the national CIHO policy, the County Health Department must define regulatory powers by regularly monitoring, evaluating, and reviewing performance data captured by the information system.

To actually promote collaboration between hospitals and primary healthcare institutions, shared savings must explicitly reward hospitals for savings or be applied to organizational forms such as CIHOs. Although the findings from this study demonstrated that a proportion of care has been moved from hospital to THCs, the anticipated cost savings may not be assured, similar to the conclusion drawn from another study [23] in England. So far, there has been no evidence of observed savings at the CIHO level; consequently, proper incentives that reward integrations at the clinical and service levels are not in place.

Findings from Deqing cannot be automatically generalized to the rest of the CIHOs in Zhejiang Province, since each one varies in scale and maturity, despite sharing the same priorities. Apart from the CIHOs that take the lead in development like Deqing, the majority in the rest of the counties are still at their provider-alignment development stage. More commonly, payment reforms that match CIHO policy are not in place or fully implemented yet for those underdeveloped counties.

Moreover, the CIHO pilot project is still in its early stage (having been implemented for less than three years), due to the lack of evidence of claims-based data. Thus, it is too early to determine whether or which specific aspects of the integrated delivery model will result in superior patient outcomes. From the perspective of IDS evaluation, emphasis is mainly placed on its role in reducing costs and improving quality of care [24,25]. There is much evidence on the positive effects of IDS on care quality and service effectiveness [26,27] not only in specific disease groups but also on disease prevention [28]. It would be worthwhile for future research to analyze the influence of CIHOs on the costs, quality, and experience of care over the following years.

## Conclusion

After one year of organizational integration, administrative monitoring data from the county and organization level show that Deqing CIHO has progressed in terms of reducing hospitalizations outside of the network and capacity building in THCs, with the potential of not only facilitating an ordered and hierarchical referral system but also controlling health expenditure. However due to the absence of a control group in our research design, the three-year time trend does not necessarily prove a cause-and-effect association. Further evaluation is required to assess the impacts on the quality and experience of care that are estimated based on claim-based data at individual level.

## Data accessibility statement

The datasets generated during and/or analysed during the current study are available from the corresponding author on reasonable request.
